# Disentangling fatigue from anhedonia: a scoping review

**DOI:** 10.1038/s41398-020-00960-w

**Published:** 2020-08-07

**Authors:** Ruel R. Billones, Saloni Kumar, Leorey N. Saligan

**Affiliations:** National Institute of Nursing Research, National Institutes of Health, Bethesda, MD USA

**Keywords:** Diseases, Schizophrenia

## Abstract

Fatigue and anhedonia are commonly reported, co-occurring clinical symptoms associated with chronic illnesses. Fatigue is a multidimensional construct that is defined as a distressing, persistent, subjective sense of physical, cognitive, or emotional tiredness that interferes with usual functioning. Anhedonia is a component of depressive disorders and other psychiatric conditions, such as schizophrenia, and is defined by the reduced ability to experience pleasure. Both symptoms greatly affect the health-related quality of life of patients with chronic illnesses. Although fatigue and anhedonia are commonly associated with each other, understanding the differences between the two constructs is necessary for diagnosis and clinical treatment. A scoping review was conducted based on published guidance, starting with a comprehensive search of existing literature to understand the similarities and differences between fatigue and anhedonia. An initial search of PubMed using fatigue and anhedonia as medical subject headings yielded a total of 5254 articles. A complete full-text review of the final 21 articles was conducted to find articles that treated both constructs similarly and articles that presented fatigue and anhedonia as distinct constructs. About 60% of the reviewed articles consider both constructs as distinct, but a considerable number of the reviewed articles found these constructs indistinguishable. Nomenclature and biology were two themes from the reviewed articles supporting the idea that anhedonia and fatigue are indistinguishable constructs. The information generated from this review is clinically relevant to optimize the management of fatigue related to anhedonia from other fatigue subtypes.

## Introduction

Fatigue is a commonly reported and disabling symptom in neurological^1,2^, rheumatologic^3,4^, and oncologic conditions^5^. It is generally defined as a feeling of malaise, lack of energy, and complaints of tiredness and exhaustion^6^. Fatigue is often associated with depression and is described as a core symptom of major depressive episode (MDE)^7,8^. However, one study reported that fatigue independently decreased the quality of life of patients when controlling for depression and other symptoms^6^. Despite its prevalence, the etiology of fatigue is still poorly understood due to the multidimensionality of this construct^9^. Fatigue is proposed to comprise of physical, affective, and cognitive dimensions^10,11^. Currently, fatigue is measured by validated self-report instruments^12^.

Anhedonia is also present in a myriad of diseases; however, it is most commonly known as a major symptom of depression^13^ and other psychiatric conditions, such as schizophrenia^7,14^. Anhedonia, which translates to “without pleasure” in Greek^15^, is defined as the inability or diminished ability to experience pleasure^16^. Like fatigue, anhedonia has been proposed to comprise of different categories of pleasure, such as physical, social, anticipatory, motivational, and consummatory pleasures. Individuals with anhedonia are still able to experience pain and negative emotions, but usually they no longer participate in previously pleasurable activities^17^. Like fatigue, measuring anhedonia is difficult as it relies on the subjective experience of pleasure and is generally measured by self-report.

Seminal articles have explored the relationships of fatigue and anhedonia in psychiatric conditions. For example, endogenomorphic depression equated low energy to fatigue and anhedonia without distinguishing one from the other^18^. In schizophrenia, although there was also no distinction of low energy as fatigue or anhedonia, the understanding of low energy was linked to a neurological dysfunction in this disorder^19^. According to the Diagnostic and Statistical Manual of Mental Disorders (DSM-V), anhedonia and fatigue are features of psychiatric disorders, most notably, major depressive disorder (MDD) and schizophrenia^7^. While the two symptoms are commonly understood as co-occurring within these psychiatric conditions, current research elucidates the individual complexities and unique attributes of each symptom^20,21^. The recommended management of fatigue and anhedonia involves pharmacological stimulants and treatment of the associated chronic illness^22^. Despite these advances, there is still limited literature that clarifies the relationship between fatigue and anhedonia. This scoping review was conducted based on established methodological frameworks^23^ in order to clarify the relationship between fatigue and anhedonia from the extant literature.

## Methods and study selection

An initial literature query was conducted using the PubMed database with the assistance of a medical librarian at the National Institutes of Health. We used “fatigue” and “anhedonia” as MeSH terms and included the corresponding synonyms, such as “lassitude” and “tiredness.” This initial search yielded 5254 articles.

Fatigue and tiredness are used interchangeably. For example, in some measures like the Fibromyalgia Impact Questionnaire (FIQ), its main descriptor is fatigue, and then one of its subscale is morning tiredness^65^. In this case, fatigue and tiredness are loosely differentiated. This non-differentiation is also observed in the way fatigue is described in many medical conditions where it states, “the tiredness or lack of energy in fatigue is associated with exhaustion”^66^. For this review, the authors focused solely on fatigue as the construct of interest and its association with anhedonia. After removing “lassitude” and “tiredness” as search terms, 117 articles remained.

Our second filtering criteria aimed to remove articles that were not written in English, did not enroll human participants, or were published before 2008. Letters, commentaries, meeting abstracts, editorials, and dissertations were also excluded. This second screen narrowed our initial yield total to 61 articles for full-text review.

**Fig. 1:**
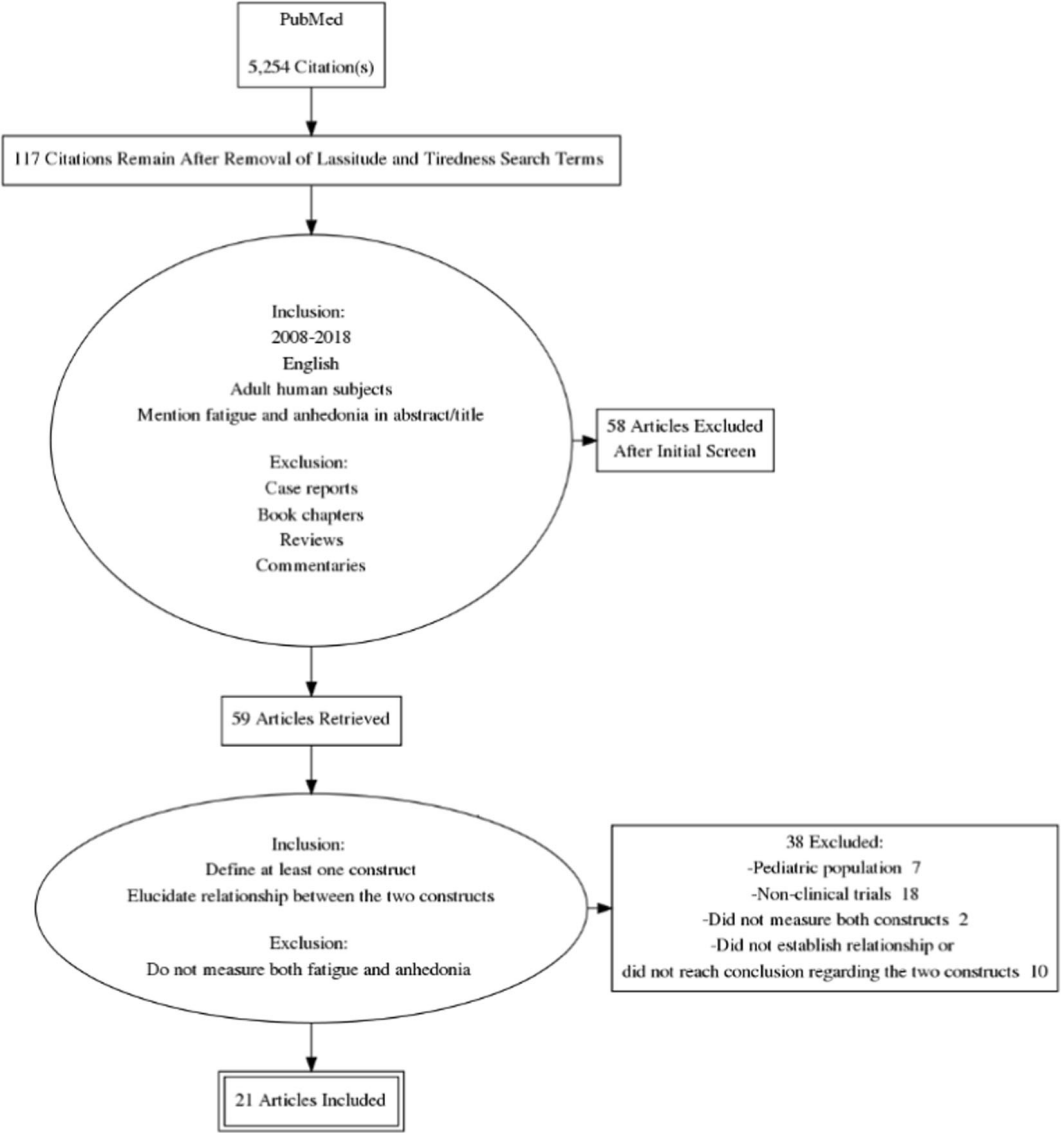
Screening flow process. Figure describes the procedures and the eligibility criteria used to select articles to be included in the review, based on the methods required for scoping reviews^67^.

To establish the relationship between the two constructs, articles that did not measure both fatigue and anhedonia were excluded. Further, articles that did not define at least one of the constructs or help elucidate the relationship between the two constructs were also excluded. A final 21 articles were included in this scoping review (Fig. 1). These 21 articles are summarized in Tables 1 and 2.

**Table 1:** Fatigue and anhedonia as associated constructs.

Author	Population	Definition of fatigue	Measurement of fatigue	Definition of anhedonia	Measurement of anhedonia	Relationship of the constructs
Capuron et al.^24^	*n* = 35 hepatitis C virus (HCV) patients	Not specified; fatigue subscales tested: general, physical, mental, reduced activity, reduced motivation	MFI	Not specified	SHAPS	Cytokines may contribute to anhedonia and fatigue in depression due to effects on dopamine function. Reduced activation of the ventral striatum was significantly correlated with greater symptoms of anhedonia, depression, and fatigue
DellaGioia et al.^25^	*n* = 10 healthy participants	Not specified	POMS-Fatigue	Social interest	MADRS-Anhedonia	Bupropion (norepinephrine and dopamine reuptake inhibitor) had NO effect on lipopolysaccharide-induced depressive symptoms (anhedonia and fatigue). This suggests that these symptoms can be reduced by serotonin reuptake inhibition (Hannestad et al.^46^) but not by norepinephrine or dopamine reuptake inhibition
Hannestad et al.^46^	*n* = 11 (men = 6) healthy patients	Not specified	MADRS-Lassitude	Social anhedonia: visual analog scale of “I want to be alone” versus “I want to be with other people”	VAS	Citalopram, which has negligible effects on neurotransmitters other than serotonin, can reduce endotoxin-induced fatigue. Most of the preventative effect of citalopram was due to an inhibition of endotoxin-induced increases in the lassitude item of MADRS, which measures fatigue and motivation
Johansson et al.^29^	*n* = 627 (age range: 65–82)	Loss of energy	SF-36	Loss of interest in usual activities	HADS	Fatigue, anhedonia, and sleepiness were amalgamated into the same symptom cluster called “sickness behavior.” This symptom cluster is associated with inflammation
Pfeil et al.^33^	*N* = 218 unemployed (at least 12 months) patients at least 50 y/o	Loss of energy	DSM-IV TR or DSM-V criteria; PHQ-9	Loss of interest and pleasure	DSM-IV TR or DSM-V criteria; PHQ-9	Depressed mood, anhedonia, and fatigue are the most frequently reported symptoms in patients with minor depression. These symptoms distinguish minor depression from major depression
Chaudhari et al.^68^	*n* = 267 PD patients (*n* = 89 control/placebo) (men = 58)	“Fatigue (tiredness) or lack of energy”—as defined by the NMSS	NMSS	“Difficulty experiencing pleasure”—as defined by the NMSS	NMSS	Rotigotine transdermal system has a positive effect on fatigue and mood disturbances (symptoms of depression and anhedonia), and apathy in patients with PD
Solla et al.^37^	*n* = 81 PD patients (men = 48)	Physical fatigue. Classified as a non-motor symptom	PFS; FSS	Classified as a non-motor, affective symptom	NMSS	Motor and non-motor symptoms of PD are related to fatigue severity, although non-motor symptoms and mostly affective conditions (anhedonia) and sleep disturbances were the main factors influencing fatigue. The affective sphere seems to be closely related to the appearance of fatigue
Tsai et al.^39^	*n* = 986,647 veterans from 130 VA facilities (men = 897,849)	Generally accepted to be a somatic symptom	PHQ-9	Not known to be somatic or non-somatic. Different models argue differently	PHQ-9	DSM-5 MDD symptoms are best represented by two factors, as somatic and non-somatic (or affective) factors. Somatic factors include symptoms like fatigue and anhedonia

Lists the articles that presented fatigue and anhedonia as indistinguishable constructs.

**Table 2:** Fatigue and anhedonia as separate constructs.

Author	Population	Definition of fatigue	Measurement of fatigue	Definition of anhedonia	Measurement of anhedonia	Relationship of the constructs
Ang et al.^32^	*n* = 505 healthy (men = 211, 23 = gender undisclosed); online survey	The feeling of exhaustion caused by the exertion of effort, which is unrelated to actual exertion of energy by muscles	MFIS	Is a mood disorder characterized by an inability to derive pleasure	SHAPS	Different subtypes of apathy are predictive of different associations with depression, anhedonia, and fatigue in healthy people. Apathy and anhedonia have a close relationship; however, there are unique aspects of anhedonia not related to apathy
Bennett et al.^40^	All women (*n* = 20 CFS, *n* = 20 post cancer fatigue, *n* = 16 major depression)	Physical fatigue in chronic fatigue: “difficulty achieving motor tasks,” mental fatigue in chronic fatigue: “difficulty achieving cognitive tasks”	SCIN; SPHERE	“Loss of motivation”, “mood disturbance”	SCIN, SPHERE	Fatigue in women with chronic fatigue syndrome, post-infective fatigue syndrome, post-cancer fatigue, and major depression is an indistinguishable symptom of depression. Anhedonia, however, is unique and a distinguishable symptom of depression. Fatigue–sadness was most associated with an increased risk of major cardiac events
Doyle et al.^41^	*n* = 408 hospitalized with acute coronary syndrome (ACS) (men = 326.7)	Classified as vital exhaustion, “tired,” “without energy”	HADS-D; BDI-FS	Not specified	HADS-D; BDI-FS	Anhedonia may be potentially less cardiotoxic than fatigue and sadness, which are strong predictors of cardiovascular prognosis
Drijgers et al.^45^	*n* = 25 with PD, 25 healthy controls (AGE matched) female = 2 in each group, 4 total	Not specified	POMS	A mood and motivational symptom	SHAPS	Acute stimulation of methylphenidate (dopamine reuptake inhibitor), but not pramipexole (dopamine 2 receptor agonist), improved anhedonia and vigor in PD patients, implies that dopamine plays a role in mood (anhedonia) but not cognition (fatigue)
Emmert-Aronson and Brown^27^	*N* = 2907 anxiety/mood disorder patients	As defined by DSM-IV	ADIS-IV-L-semi-structured interview and dimensionally rated	Correlated with low mood, as defined by DSM-IV	ADIS-IV-L-semi-structured interview and dimensionally rated	“Depressed mood” and “anhedonia” showed higher discrimination of higher depression (alpha = 3.25) than the other symptoms. Fatigue also discriminates better than most symptoms (alpha = 1.97). Anhedonia and depressed mood are required for diagnosis of MDD but could also create a very inclusive and encompassing symptom set for all severities of depression that includes depressed mood, anhedonia, fatigue, and concentration difficulties
Hawkins et al.^28^	*n* = 326 HF patients (women = 132)	Physical, somatic symptom of depression	PHQ-9	Behavioral, non-somatic symptom of depression	PHQ-9	Interventions that target a patient’s somatic symptoms (fatigue) may not yield maximum cognitive benefit compared to comprehensive treatment that targets depressed mood, anhedonia, and other non-somatic symptoms
Lapidus et al.^30^	*n* = 11 with major depressive disorder (MDD), *n* = 10 age-/sex-matched controls	Not specified. Noted to have an effect on motivation to participate in enjoyable activities	MFI	Reduced capacity to experience pleasure	QIDS-SR	There is no association between fatigue and glutathione levels (antioxidant) in the MDD group. Anhedonia is negatively correlated with brain occipital glutathione levels, supporting the role of glutathione in oxidative stress and inflammation, specifically in anhedonia in MDD
Leventhal et al.^44^	*n* = 187 regular smokers	Fatigue domain in POMS: “Fatigue (e.g., “worn out,” “fatigued”)”	POMS-Fatigue	Anhedonia, a specific factor for depression indicative of reduced interest/motivation, pleasure, and positive affect, and is putatively distinct from anxiety	MASQ-AD; (e.g., “felt like there wasn’t anything interesting or fun to do,” “felt like nothing was very enjoyable”)	Anhedonia, but not fatigue, was associated with only abstinence-induced reductions in positive affect. Used the tripartite model to elucidate the relation of anxiety and depressive symptoms to tobacco withdrawal and found that anhedonia predicts greater smoking relapse risk
McGuire et al.^31^	*n* = 323 (women = 73) patients who were admitted because of a cardiac event	Physical or mental weariness, part of somatic symptom cluster of depression	BDI; HRSD	Loss of interest or pleasure in activities, part of cognitive/affective symptom cluster of depression	BDI, HRSD	Cognitive/affective symptom clusters (anhedonia) is important for initial screening and are unique correlates of depression in patients with CHD. However, cognitive/affective symptoms must accompany somatic symptoms (fatigue) before depression can be diagnosed
Olivan-Blazquez et al.^42^	*n* = 741 primary care patients	Loss of energy within the context of depression	DSM-V criteria; CIDI v2	Decreased/lack of interest within the context of depression	DSM-V criteria	Out of the nine proposed diagnostic symptoms of depression, anhedonia and depressed mood are essential for the diagnosis. Anhedonia was significant in the first 6–12 months of onset of depression. Fatigue was only significant within the first 12 months of onset of depression
Ritchie et al.^35^	*n* = 1000 community-dwelling older adults (age >65 years)	Feeling tired, classified as a physical symptom	BSS	Classified as an affective symptom	BSS	Fatigue was found to be most related to other symptoms and most commonly endorsed when evaluating symptoms in older adults. No conclusion on anhedonia
Sibitz et al.^36^	*n* = 290 physically ill general hospital patients	Decreased energy or increased fatigability	PQm (adapted)	Loss of interest of pleasure	PQm (adapted)	Inclusion of “fatigue” does not yield an advantage to just using “depressed mood” and/or “anhedonia” to screen depression. Special attention to be paid to “anhedonia” as a screening symptom of depression
Trincas et al.^38^	*n* = 456 participants (women = 328), with dysphoric reactions (low mood to depression)	Physical symptom of depression	CES-D; DSM-V criteria	Predicted by Failure schema in the Young Schema Questionnaire, which is the belief that one is inadequate compared with others	CES-D; DSM-V criteria	Depression is not a unitary phenomenon. Fatigue and other physical symptoms were not predicted by any of the early maladaptive schemas (EMSs). The occurrence of anhedonia was predicted by the EMS Failure, that is, the belief that one is inadequate compared with others

Lists the articles that presented fatigue and anhedonia as distinct constructs.

## Results

### Characteristics of the selected articles

Sixteen of the 21 articles were published in the past 5 years. More than half (60%) were cross-sectional studies^24–40^, while two were longitudinal prospective cohort studies^41,42^. Two articles used both cross-sectional and longitudinal study designs^1,24,40,43^. There was one within-subject experimental study design^44^ and three randomized placebo-controlled trials^25,45–47^.

Across the 21 studies, 9 were conducted in Europe^26,29,32,33,36–38,41,42,45^, and 10 were conducted in the United States^24,25,27,28,30,31,35,39,44,46^. One study took place in Australia^40^, and another study was a multi-continental venture involving 12 different countries^47^. Out of the 9175 participants enrolled across these studies (excluding the veteran population study), 45.7% of the participants enrolled were male. One study analyzed medical records from a veteran database that enlisted close to a million veterans in the United States, 91% of whom were male^39^. Three studies enrolled in-patient participants^31,36,41^, and seven studies enrolled outpatient populations^24,26,30,36,37,42,45^. The rest enrolled healthy or primary care patients.

A common patient population studied was those with heart conditions (19%), including depression in acute coronary syndrome patients^41^, heart failure^28^, coronary heart disease^31^, and impaired cardiac function in community-dwelling elders^29^. About 14% of the studies enrolled patients with Parkinson’s disease (PD)^37,45,47^. Other patient populations included patients with chronic hepatitis C virus (HCV)^24^, cancer^40^, anxiety and mood disorders^27^, MDD or depression^30,33^, dysphoric episodes^38^, and non-psychiatric conditions who were hospitalized^36^. Healthy volunteers^25,32,46^ and primary care patients^42^ were also assessed, as were community-dwelling elder adults^35^ and tobacco smokers^44^.

Two studies collected data via online assessments^32,38^, and four studies either retrospectively analyzed data or utilized previously collected datasets^29,31,39,47^. Two studies utilized healthy controls^30,45^, and six studies used the control groups or placebo groups for comparison^24–26,33,46,47^.

### Assessments of fatigue and anhedonia

Questionnaires used in the reviewed articles that assessed both fatigue and anhedonia included the Clinical Interview Neurasthenia, the Somatic Psychological Health Report, Hospital Anxiety and Depression Scale, Beck Depression Inventory, Hamilton Rating Scale for Depression, Diagnostic and Statistical Manual of Mental Disorders (DSM-IV and DSM-V), Brief Symptom Screen, modified Patient Questionnaire, the Center for Epidemiologic Studies Depression Scale, the Montgomery–Asberg Depression Rating Scale (MADRS), Non-Motor Symptoms Questionnaire, and the Patient Health Questionnaire (PHQ-9).

The DSM-IV and DSM-V criteria were used the most by the reviewed articles to define fatigue^27,33,38,42^. A total of seven different questionnaires were used to specifically assess fatigue, which included the Modified Fatigue Impact Scale, Multidimensional Fatigue Inventory, Profile Mood of States-Fatigue (POMS-F), Vitality Scale of the Short Form Health Survey (SF-36), Fatigue Severity Scale, Parkinson’s Disease Fatigue Scale, and the sleep/fatigue domain of the Non-Motor Symptoms Scale (NMSS).

The Snaith–Hamilton Pleasure Scale was the commonly used questionnaire to assess anhedonia^24,32,45^ and has been validated to measure anhedonia^48^. Other questionnaires that were used to assess anhedonia included the Visual Analog Scale for anhedonia, the Quick Inventory of Depressive Symptomology-Self Report, Mood and Anxiety Symptom Questionnaire—Anhedonic Depression Symptom, item 8 of the MADRS-Anhedonia, and the mood/apathy domain of the NMSS.

### Definition of fatigue

There was no consensus on the definition of fatigue. By far, the most common definition of fatigue described it as a loss of energy or a lack of energy^29,33,36,42^. Similarly, fatigue was also described as a physical feeling of lassitude^46^, feeling tired^35^, the feeling of exhaustion or vital exhaustion^32,41^, worn out as described by POMS^44^, and decreased energy or increased fatigability^36^. Some articles classified fatigue as a non-motor symptom of PD^26,37^, while other articles classified it as a somatic or physical symptom of depression^28,31,35,38,39^. Capuron et al.^24^ tested different subscales of fatigue, including general fatigue, physical fatigue, and mental fatigue. Bennett et al.^40^ described both physical and mental fatigue, where physical fatigue in chronic fatigue syndrome was defined as “difficulty achieving motor tasks” and mental fatigue was defined as “difficulty achieving cognitive tasks.”

### Definition of anhedonia

There was not a clear consensus on the definition of anhedonia. By far, the most common definition of anhedonia was a loss of interest or pleasure in activities^29,31,33,36^. Similarly, anhedonia was also defined as a decreased/lack of interest^42^, dysphoria or a markedly diminished interest/pleasure in most daily activities^34^, and reduced interest/motivation, pleasure, and positive affect and is putatively distinct from anxiety^44^. Anhedonia was also defined as an impaired ability to experience pleasure or difficulty experiencing pleasure^24,30,47^. The construct of anhedonia was categorized as a behavioral, non-somatic symptom of depression^28^ or classified as a non-motor, affective symptom in PD^37^ or an affective symptom in community-dwelling older adults^35^. Anhedonia was also commonly classified as a mood disturbance or motivational symptom^32,40,45^. Anhedonia was also defined by its dimensions, such as social anhedonia, which was assessed by a one-item VAS, “I want to be alone” on one end and “I want to be with others” on the other end^46^. Trincas et al.^38^ noted that anhedonia was predicted by the *Failure* schema in the Young Schema Questionnaire, which is the belief that one is inadequate compared with others in the context of depressive symptoms following dysphoric episodes.

### Relationship of fatigue and anhedonia

Eight studies presented fatigue and anhedonia as associated or overlapping constructs. For example, four articles presented fatigue and anhedonia both as symptoms of depression^24,33,39,46^. These articles showed the two constructs as related within the context of depression. Three articles presented potentially common mechanisms or pathways to explain how the symptoms of fatigue and anhedonia are associated^24,46,47^. Capuron et al.^24^ presented a potential mechanism by which inflammatory cytokines affect fatigue and anhedonia, thereby implicating dopamine function as a common mechanism shared by the two constructs in the HCV patient population. Hannestad et al.^46^ implicated citalopram, a serotonin reuptake inhibitor, as having a preventative effect on endotoxin-induced increases in fatigue and motivation (anhedonia). Chaudhari et al.^68^ found that the rotigotine transdermal system (a non-ergoline dopamine receptor agonist) may have a positive effect on fatigue and mood disturbances (anhedonia) in patients with PD. Fatigue and anhedonia when amalgamated into a symptom cluster called “sickness behavior,” were also shown to be associated with inflammation and directly affected by impaired cardiac function in patients with cardiac failure^29^. Finally, two articles presented fatigue and anhedonia as one of the prodromal symptoms of a condition, such as early-stage PD^26,37^.

However, 12 articles presented fatigue and anhedonia as separate constructs, each with its own unique implications. Four articles presented anhedonia as a unique and distinguishable symptom of depression in that fatigue alone is not enough to diagnose major depression^31,36,40,42^. These four articles described the importance of anhedonia, more so than fatigue, as a diagnostic screening tool for depression, because it was found that anhedonia is an essential and distinguishing feature of a depression diagnosis. Two articles implicated separate pathophysiological mechanisms for fatigue and anhedonia^30,45^. Drijgers et al.^45^ found that acute stimulation of methylphenidate (norepinephrine–dopamine reuptake inhibitor) improved anhedonia and vigor in PD patients, implying that dopamine plays a role in anhedonia (mood) but not fatigue (cognition). Lapidus et al.^30^ found that anhedonia, but not fatigue, is negatively correlated with brain glutathione levels in patients with MDD. Three other articles found that either fatigue or anhedonia had unique implications on specific patient populations^28,41,44^. Doyle et al.^41^ found that anhedonia could potentially be less cardiotoxic than fatigue and sadness in hospitalized patients with acute coronary syndrome. Hawkins et al.^28^ found that interventions targeting patients’ somatic symptoms, such as fatigue, may not yield maximum cognitive benefit in comparison to comprehensive treatment targeting non-somatic symptoms, such as anhedonia in patients with heart failure. Leventhal et al.^44^ implicated anhedonia, but not fatigue, as being predictive of greater smoking relapse risk in the tobacco-smoking population.

Anhedonia was found to be predicted by an early maladaptive schema of *failure* when analyzing symptoms of depression that emerged after dysphoric episodes and its potential to be predicted by stable personality characteristics like early maladaptive schemas^38^. In aging populations (age >65 years), fatigue but not anhedonia was found to be most related to other symptoms and most commonly reported by the elderly population^35^. Finally, fatigue and anhedonia were also shown to be associated with different subtypes of apathy in healthy people^32^.

## Discussion

This review explored the association between fatigue and anhedonia. About 60% of the reviewed articles considered both constructs as distinct, but still, a considerable number of the reviewed articles found these constructs indistinguishable. This finding has several clinical and research implications: (1) the lack of agreement in the association of both constructs poses more questions on current diagnostic criteria that clumps both constructs together, (2) the initial evidence of distinct biologic underpinnings for each construct provides opportunities to develop targeted therapies, and (3) the lack of consensus in a standard approach to measuring these constructs provides challenges to appropriately apply the diagnostic criteria and evaluate distinct etiologies for these constructs.

### Anhedonia and fatigue as indistinguishable constructs

In the reviewed articles, nomenclature and biology were two themes supporting the idea that anhedonia and fatigue are indistinguishable constructs. Some conditions, such as depression, historically consider fatigue and anhedonia as inter-related and interchangeable constructs, except when motor systems are affected. Depressive symptoms are often categorized into somatic and non-somatic symptoms^39^. Anhedonia is clustered together with concentration difficulties and psychomotor agitation/retardation; while fatigue, together with sleep difficulties, and appetite changes are often classified under the somatic category of depressive symptoms.

Anhedonia was first featured in the DSM and the International Classification of Diseases in 1992, while fatigue was featured in DSM-III in 1980^49^. In the early versions of the DSM, fatigue was suggested to mean physical energy^50^ and described as “flu-like symptoms” in depressive disorders of DSM-III^51^. There were no diagnostic criteria for fatigue as a stand-alone somatoform disorder in DSM-III^51^. Hence, fatigue, like insomnia, was less frequently diagnosed as a separate condition because the established criteria prohibited the classification of either of these symptoms as a stand-alone somatoform disorder^52^.

As the historical usage of fatigue suggests a loss of physical energy, fatigue has been considered as synonymous with a loss in pleasure experience^16^. Researchers argue that loss of energy or loss of pleasure can be traced to neural systems that block reward reinforcement from the source of pleasurable stimuli^18^. When the DSM was revised, the term anhedonia expanded from the “loss of pleasure” to also include the loss of interest and/or capacity for pleasure with the inclusion of melancholia features, which is the inability to respond to all pleasurable stimuli^53^. It was from this distinction in DSM-IV that fatigue meshed with the melancholia construct^53^.

Another concept that clumps these two constructs together is sickness behavior. Sickness behavior refers to the coordinated set of behavioral changes that develop in individuals during the course of an infection. These changes are due to the effects of pro-inflammatory cytokines that influence the nervous system and behavior^54^. In depression, a network of cytokines are believed to regulate mood and influence motivation^54^. It is believed that, during acute infection, pro-inflammatory cytokines like interleukin (IL)-1, IL-6, and tumor necrosis factor-alpha are released peripherally via a fast transmission pathway, which influences the activities of the primary afferent nerves. The interactions between the pro-inflammatory cytokines and the primary afferent nerves activate the hypothalamic–pituitary–adrenal axis producing a constellation of behavioral symptoms referred to as sickness behavior, which include fatigue and anhedonia^54,55^. What is lacking in this hypothesis is that these specific symptoms exist under a wider cluster of comorbid conditions so that these sickness behaviors may not exclusively include fatigue and anhedonia behaviors. Long-term administration of inflammatory cytokines was also observed to decrease activation in the ventral striatum, which significantly correlated with greater symptoms of anhedonia, depression, and fatigue^24^. Administration of these inflammatory cytokines was associated with decreased presynaptic striatal dopamine, and these changes correlated with behavioral alterations in fatigue and anhedonia^47^. Application of a non-ergoline dopamine receptor agonist (rotigotine transdermal patches) improved both fatigue and anhedonia in patients with PD. These findings support the idea that the interplay of peripheral and central mechanisms triggered by inflammation blur distinct pathways that can distinguish fatigue from anhedonia.

### Anhedonia and fatigue as distinct constructs

Clinical reports of persistent fatigue after remission of depression helped pave the notion that perhaps anhedonia and fatigue are distinct constructs^32^. The reviewed articles proposed that distinct biology distinguishes these constructs. The roles of dopamine^47^, serotonin^25^, and oxidative stress/inflammation^30^ were specifically mentioned as pathways that can distinguish fatigue and anhedonia.

Methylphenidate (dopamine reuptake inhibitor) was used to improve anhedonia but not fatigue in patients with PD in comparison to controls^45^. Pramipexole (a dopamine D2 receptor agonist), on the other hand, had negative effects on mood and fatigue but had an insignificant effect on anhedonia^46^. Pretreatment of bupropion (a dopamine and norepinephrine reuptake inhibitor) had no significant effect on lipopolysaccharide-induced anhedonia and fatigue, while pretreatment with citalopram (a serotonin reuptake inhibitor) significantly improved endotoxin-induced fatigue but had no effect in anhedonia^46^). These findings serve to implicate the distinct roles of dopamine and serotonin receptors in distinguishing anhedonia from fatigue.

### Fatigue and anhedonia in the context of psychiatric conditions

Fatigue and anhedonia have been explored not only in depression but also in other psychiatric conditions such as schizophrenia, where one in every three patients has anhedonia^14,56–58^. In depression, the seminal work of Klein^18^ pointed out the construct of low energy in depression, specifically in individuals with the depressive-neurotic type of personality. This personality type has low self-esteem and depends on external environmental cues to experience pleasure^18^. In schizophrenia, anhedonia is a core negative symptom and is associated with functional impairment^59^. It is captured as the “diminished ability to experience pleasure”^19^.

Instruments used to assess depression used similar and distinguishing terms to capture fatigue and anhedonia. For example, MDD, as described in DSM-V, treats fatigue as “low energy” and anhedonia as a “lack of pleasurable interests.” In the 17-item Hamilton Depression Scale, the construct of fatigue is captured in items that used the following terms: “somatic symptoms,” “loss of energy,” and “fatigability,” while anhedonia is captured using the following terms: “loss of interest in hobbies, decreased social activities, decreased in productivity, and inability to work”^60^.

### State versus trait anhedonia

In differentiating fatigue from anhedonia, the understanding of state versus trait anhedonia may be helpful. Anhedonia is a complex and nuanced symptom related to a number of health conditions. It occurs independently of one’s ability to experience general stimulation and negative emotions^61^. In addition, it can occur with or without feelings of sadness^62^. The pathological symptom of anhedonia presents itself in various psychological and physical conditions and likely involves dysregulation of a number of neurochemical pathways in the brain^63^. When other mental or physical symptoms are present, anhedonia can be conceptualized as a pathologic symptom (state) of the primary condition, such as in depression or schizophrenia. Nevertheless, the literature is silent on whether state or trait anhedonia is associated with fatigue. However, hedonic inability, whether as a symptom or a trait, is frequently correlated with the presence of psychomotor delay even if confused with affective flattening in schizophrenia or even if it represents as a psychopathologic characteristic that is specific for depression^64^.

### Gaps in the study

Three gaps were observed in this review: (1) lack in consensus in the definitions of fatigue and anhedonia, (2) lack of agreement in standard measures to assess fatigue and anhedonia, and (3) no validated shared and/or distinct pathways that can explain the association of both constructs. The lack of consensus stems from how the fatigue construct was understood within depression in DSM-III and the way fatigue has evolved to its current stand-alone construct in DSM-V to include its dimensions, such as physical, cognitive, and behavioral components^7^.

Anhedonia continues to be a diagnostic criterion in MDE diagnosis in DSM-V separate from fatigue. Its description overlapped and became more pronounced in the diagnostic criteria of unspecified depressive disorder with melancholic features^7^. However, since the anhedonia construct has evolved to include dimensions of perception (cognitive), action/sensorial/motor (physical), and pleasure (affective), it began to overlap with the construct of apathy (lack of emotion). These fatigue and anhedonia usages were first treated as co-existing constructs in depression. The comorbid status of depression with other medical conditions impacts the understanding of the fatigue–anhedonia relationship. One example of this is reflected in PD with comorbid depression. The lack of condition-specific definitions of constructs are observed in the current measures that assess anhedonia and fatigue. Further, the lack of clarity in the relationship of fatigue and anhedonia has slowed our understanding of the neural pathways that influence these behaviors, including the role of presynaptic striatal dopamine, the serotonergic regulation, oxidative stress pathways, and the role of inflammation.

### Recommendations

There is a need to establish condition-specific definitions to understand further the dynamic relationships of fatigue and anhedonia. In the future revision of the DSM criteria, the dimensional construct of fatigue should be considered to classify its severity, duration, and frequency. Similarly, the melancholic features of depressive disorders require a dimensional understanding of anhedonia as a construct. Future research designs must capture the dimensional understanding of fatigue and anhedonia in relation to specific medical conditions of interest.

## Conclusion

The purpose of this review was to clarify the relationship of fatigue and anhedonia. Historically, diagnostic nomenclature of fatigue and anhedonia had overlaps in its usage (DSM-III, DSM-IV). Through time, anhedonia had its own descriptive criteria in the melancholia features of MDDs. In DSM-V, fatigue severity is recognized as a unique entity. The future of discriminating the relationships between the two constructs must take into account the health condition being studied and its comorbidities.

## References

[1] Friedman JH (2007). Fatigue in Parkinson’s disease: a review. Mov. Disord..

[2] Krupp L (2006). Fatigue is intrinsic to multiple sclerosis (MS) and is the most commonly reported symptom of the disease. Mult. Scler..

[3] Murphy SL, Smith DM, Clauw DJ, Alexander NB (2008). The impact of momentary pain and fatigue on physical activity in women with osteoarthritis. Arthritis Rheum..

[4] Ramsey-Goldman R, Rothrock N (2010). Fatigue in systemic lupus erythematosus and rheumatoid arthritis. PM R..

[5] Liu L (2012). The longitudinal relationship between fatigue and sleep in breast cancer patients undergoing chemotherapy. Sleep.

[6] Visser MR, Smets EM (1998). Fatigue, depression and quality of life in cancer patients: how are they related?. Support Care Cancer.

[7] American Psychiatric Association. *Diagnostic and Statistical Manual of Mental Disorders* 5th edn (APA, Washington, DC, 2013).

[8] Ray C (1991). Chronic fatigue syndrome and depression: conceptual and methodological ambiguities. Psychol. Med.

[9] Sharpe M, Wilks D (2002). Fatigue. BMJ.

[10] Glaus A, Muller S (2001). [Measuring fatigue of cancer patients in the German-speaking region: development of the Fatigue Assessment Questionnaire]. Pflege.

[11] Haghighat S, Akbari ME, Holakouei K, Rahimi A, Montazeri A (2003). Factors predicting fatigue in breast cancer patients. Support Care Cancer.

[12] Whitehead L (2009). The measurement of fatigue in chronic illness: a systematic review of unidimensional and multidimensional fatigue measures. J. Pain Symptom Manag..

[13] Higa-McMillan CK, Smith RL, Chorpita BF, Hayashi K (2008). Common and unique factors associated with DSM-IV-TR internalizing disorders in children. J. Abnorm. Child Psychol..

[14] Horan WP, Kring AM, Blanchard JJ (2006). Anhedonia in schizophrenia: a review of assessment strategies. Schizophr. Bull..

[15] Fuhrer R, Wessely S (1995). The epidemiology of fatigue and depression: a French primary-care study. Psychol. Med..

[16] Berrios GE, Olivares JM (1995). The anhedonias: a conceptual history. Hist. Psychiatry.

[17] Ho N, Sommers M (2013). Anhedonia: a concept analysis. Arch. Psychiatr. Nurs..

[18] Klein DF (1974). Endogenomorphic depression. A conceptual and terminological revision. Arch. Gen. Psychiatry.

[19] Meehl PE (1973). Some methodological reflections on the difficulties of psychoanalytic research. Psychol. Issues.

[20] Dantzer R, Heijnen CJ, Kavelaars A, Laye S, Capuron L (2014). The neuroimmune basis of fatigue. Trends Neurosci..

[21] Gorwood P (2008). Neurobiological mechanisms of anhedonia. Dialogues Clin. Neurosci..

[22] National Comprehensive Cancer Network. Fatigue. https://www.nccn.org/patients/resources/life_with_cancer/managing_symptoms/fatigue.aspx (2018).

[23] Arksey H, O’Malley L (2007). Scoping studies: towards a methodological framework. Int. J. Soc. Res. Methodol..

[24] Capuron L (2012). Dopaminergic mechanisms of reduced basal ganglia responses to hedonic reward during interferon alfa administration. Arch. Gen. Psychiatry.

[25] DellaGioia N, Devine L, Pittman B, Hannestad J (2013). Bupropion pre-treatment of endotoxin-induced depressive symptoms. Brain Behav. Immun..

[26] Dujardin K (2014). Apathy in untreated early-stage Parkinson disease: relationship with other non-motor symptoms. Mov. Disord..

[27] Emmert-Aronson BO, Brown TA (2015). An IRT analysis of the symptoms of major depressive disorder. Assessment.

[28] Hawkins MA (2015). Cognitive function in heart failure is associated with nonsomatic symptoms of depression but not somatic symptoms. J. Cardiovasc. Nurs..

[29] Johansson P (2014). Sickness behavior in community-dwelling elderly: associations with impaired cardiac function and inflammation. Biol. Res. Nurs..

[30] Lapidus KA (2014). In vivo (1)H MRS study of potential associations between glutathione, oxidative stress and anhedonia in major depressive disorder. Neurosci. Lett..

[31] McGuire AW, Eastwood JA, Hays RD, Macabasco-O’Connell A, Doering LV (2014). Depressed or not depressed: untangling symptoms of depression in patients hospitalized with coronary heart disease. Am. J. Crit. Care.

[32] Ang YS, Lockwood P, Apps MA, Muhammed K, Husain M (2017). Distinct subtypes of apathy revealed by the Apathy Motivation Index. PLoS ONE.

[33] Pfeil S, Holtz K, Kopf KA, Hegerl U, Rummel-Kluge C (2017). Minor depression in older, long-term unemployed people seeking vocational support. BMC Psychiatry.

[34] Potvin O, Hudon C, Grenier S, Preville M (2010). Non-essential symptoms of depression and cognitive impairment no dementia (CIND) in community-dwelling elders without dysphoria or anhedonia. Int. Psychogeriatr..

[35] Ritchie CS (2013). Measuring symptoms in community-dwelling older adults: the psychometric properties of a brief symptom screen. Med. Care.

[36] Sibitz I (2010). ICD-10 or DSM-IV? Anhedonia, fatigue and depressed mood as screening symptoms for diagnosing a current depressive episode in physically ill patients in general hospital. J. Affect. Disord..

[37] Solla P (2014). Association between fatigue and other motor and non-motor symptoms in Parkinson’s disease patients. J. Neurol..

[38] Trincas R (2014). Specific dysphoric symptoms are predicted by early maladaptive schemas. ScientificWorldJournal.

[39] Tsai, J., Elhai, J. D., Pietrzak, R. H., Hoff, R. A. & Harpaz-Rotem, I. Comparing four competing models of depressive symptomatology: a confirmatory factor analytic study of 986,647 U.S. veterans. *J. Affect. Disord*. **165**, 166–169, (2014) 10.1016/j.jad.2014.04.075. 10.1016/j.jad.2014.04.075 24882195

[40] Bennett BK (2014). Characterization of fatigue states in medicine and psychiatry by structured interview. Psychosom. Med..

[41] Doyle F, Conroy R, McGee H, Delaney M (2010). Depressive symptoms in persons with acute coronary syndrome: specific symptom scales and prognosis. J. Psychosom. Res..

[42] Olivan-Blazquez B, Rubio-Aranda E, Garcia-Sanz O, Magallon-Botaya R (2016). Correlation between diagnosis of depression and symptoms present in primary care patients. Actas Esp. Psiquiatr..

[43] Barat P (2016). Inflammatory, endocrine and metabolic correlates of fatigue in obese children. Psychoneuroendocrinology.

[44] Leventhal AM, Ameringer KJ, Osborn E, Zvolensky MJ, Langdon KJ (2013). Anxiety and depressive symptoms and affective patterns of tobacco withdrawal. Drug Alcohol Depend..

[45] Drijgers RL (2012). The role of the dopaminergic system in mood, motivation and cognition in Parkinson’s disease: a double blind randomized placebo-controlled experimental challenge with pramipexole and methylphenidate. J. Neurol. Sci..

[46] Hannestad J, DellaGioia N, Ortiz N, Pittman B, Bhagwagar Z (2011). Citalopram reduces endotoxin-induced fatigue. Brain Behav. Immun..

[47] Ray Chaudhuri K (2013). Rotigotine and specific non-motor symptoms of Parkinson’s disease: post hoc analysis of RECOVER. Parkinsonism Relat. Disord..

[48] Nakonezny PA, Carmody TJ, Morris DW, Kurian BT, Trivedi MH (2010). Psychometric evaluation of the Snaith-Hamilton pleasure scale in adult outpatients with major depressive disorder. Int. Clin. Psychopharmacol..

[49] American Psychiatric Association. *Diagnostic and Statistical Manual of Mental Disorders* 3rd edn (APA, Washington, DC, 1980).

[50] Herz MI, Endicott J, Spitzer RL (1977). Brief hospitalization: a two-year follow-up. Am. J. Psychiatry.

[51] Gruber AJ, Hudson JI, Pope HG (1996). The management of treatment-resistant depression in disorders on the interface of psychiatry and medicine. Fibromyalgia, chronic fatigue syndrome, migraine, irritable bowel syndrome, atypical facial pain, and premenstrual dysphoric disorder. Psychiatr. Clin. North Am..

[52] Kroenke K (1994). Physical symptoms in primary care. Predictors of psychiatric disorders and functional impairment. Arch. Fam. Med..

[53] American Psychiatric Association. *Diagnostic and Statistical Manual of Mental Disorders* 4th edn (APA, Washington, DC, 1994).

[54] Dantzer R (2001). Cytokine-induced sickness behavior: where do we stand?. Brain Behav. Immun..

[55] Moreau M (2008). Inoculation of Bacillus Calmette-Guerin to mice induces an acute episode of sickness behavior followed by chronic depressive-like behavior. Brain Behav. Immun..

[56] Chapman LJ, Chapman JP, Raulin ML (1976). Scales for physical and social anhedonia. J. Abnorm. Psychol..

[57] Rado S (1956). The contribution of psychoanalysis to the medical study of behavior; the Freud Centenary Address. J. Nerv. Ment. Dis..

[58] Rado S (1962). On the retransformation of psychoanalysis into a medical science. Compr. Psychiatry.

[59] Horan WP, Green MF, Kring AM, Nuechterlein KH (2006). Does anhedonia in schizophrenia reflect faulty memory for subjectively experienced emotions?. J. Abnorm Psychol..

[60] Hamilton M (1960). A rating scale for depression. J. Neurol. Neurosurg. Psychiatry.

[61] Aguera-Ortiz L, Failde I, Mico JA, Cervilla J, Lopez-Ibor JJ (2011). Pain as a symptom of depression: prevalence and clinical correlates in patients attending psychiatric clinics. J. Affect Disord..

[62] Compton MT, Frank E (2011). Mental health concerns among Canadian physicians: results from the 2007-2008 Canadian Physician Health Study. Compr. Psychiatry.

[63] Der-Avakian A, Markou A (2012). The neurobiology of anhedonia and other reward-related deficits. Trends Neurosci..

[64] Lemke MR, Puhl P, Koethe N, Winkler T (1999). Psychomotor retardation and anhedonia in depression. Acta Psychiatr. Scand..

[65] Aparcio V, Ortega F, Carbonell-Baeza A, Cuevas M, Delgado-Ferndandez M, Jonatan R (2013). Anxiety, depression and fibromyalgia pain and severity. Behav. Psychol. Psicología Conductual..

[66] Sharpe M, Wilks D (2002). Fatigue. BMJ.

[67] Arksey H, O’Malley L (2005). Scoping studies: towards a methodological framework. Int. J. Soc. Res. Methodol..

[68] Chaudhuri, R., Martinez-Martin, P. & Trenkwalder, C. Rotigotine and specific non-motor symptoms of Parkinson’s disease: Post hoc analysis of recover. *Parkinsonism Relat. Disord*. **19**, 660–665, (2013) 10.1016/j.parkreldis.2013.02.018. 10.1016/j.parkreldis.2013.02.018 23557594

